# The application prospects of sacha inchi (*Plukenetia volubilis* linneo) in rheumatoid arthritis

**DOI:** 10.3389/fphar.2024.1481272

**Published:** 2024-10-17

**Authors:** Min Wang, Yin-Hong Xiang, Mei Liu, Shan Jiang, Jia-ying Guo, Xiao-yan Jin, Hui-feng Sun, Ning Zhang, Zhi-Gang Wang, Jian-xin Liu

**Affiliations:** ^1^ School of Pharmacy, Heilongjiang University of Chinese Medicine, Harbin, Heilongjiang, China; ^2^ Sino-Pakistan Center on Traditional Chinese Medicine, School of Pharmaceutical Sciences, School of Basic Medical Sciences, China-Pakistan International Science and Technology Innovation Cooperation Base for Ethnic Medicine Development in Hunan Province, Hunan University of Medicine, Huaihua, Hunan, China; ^3^ School of Pharmaceutical Sciences, University of South China, Hengyang, Hunan, China; ^4^ School of Pharmaceutical Sciences, Xinjiang medical University, Wulumuqi, Xinjiang, China

**Keywords:** *Plukenetia volubilis* linneo, sacha inchi, rheumatoid arthritis, inflammation, antioxidant, gut microbiota

## Abstract

Sacha Inchi (*Plukenetia volubilis* L) (SI) is a traditional natural medicine from tropical rainforests of Amazon region in South America. As a raw material for edible oil, it has various pharmacological effects such as antioxidant, anti-inflammatory, hypolipidemia, and blood pressure lowering, which have attracted increasing attentions of pharmacists. This has prompted researchers to explore its pharmacological effects for potential applications in certain diseases. Among these, the study of its anti-inflammatory effects has become a particularly interesting topic, especially in rheumatoid arthritis (RA). RA is a systemic autoimmune disease, and often accompanied by chronic inflammatory reactions. Despite significant progress in its treatment, there is still an urgent need to find effective anti-RA drugs in regard to safety. This review summarizes the potential therapeutic effects of SI on RA by modulating gut microbiota, targeting inflammatory cells and pathways, and mimicking biologic antibody drugs, predicting the application prospects of SI in RA, and providing references for research aimed at using SI to treat RA.

## Graphical Abstract

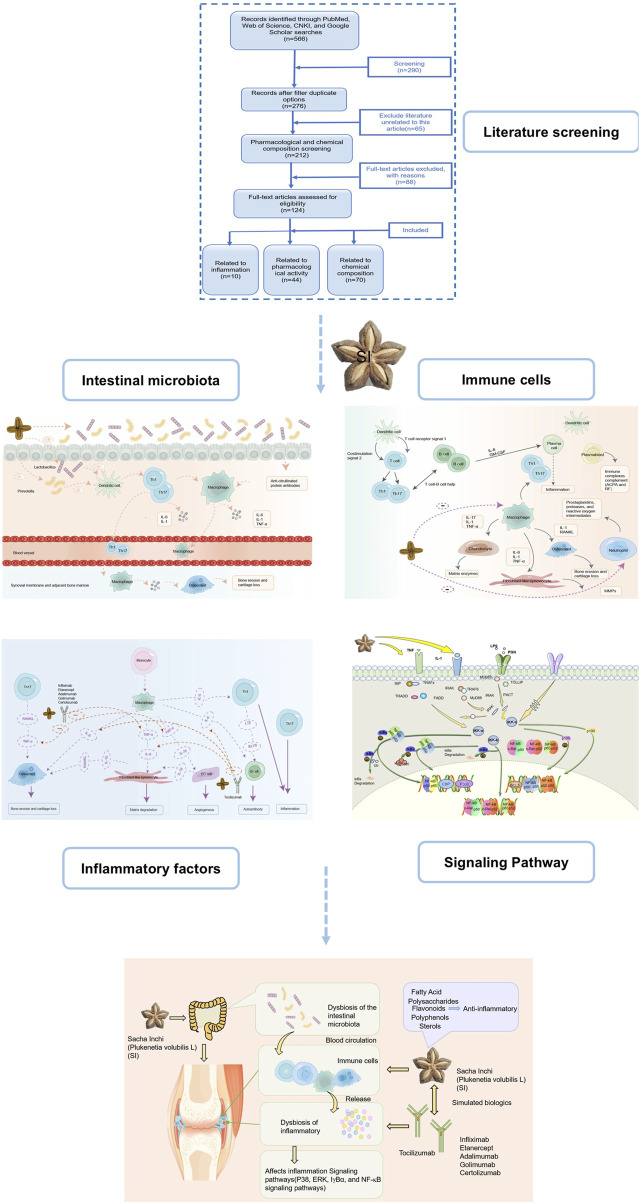

## 1 Introduction

### 1.1 Sacha inchi (SI)

Sacha Inchi (*Plukenetia volubilis* L), also known as South American oil vine, star oil vine, is a perennial woody vine plant belonging to the Euphorbiaceae family (Mhd Rodzi and Lee, 2022). It grows between altitudes of 200–2000 m and is commonly cultivated in many regions of the Amazon rainforest, such as Brazil, Peru, and Ecuador. Discovered by indigenous peoples over 3,000 years ago, it has been widely consumed as a healthy food with a long history (Lu et al., 2023). SI is rich in α-linolenic acid, vitamin E, phytosterols, tocopherols, phenolic acids and other substances (Ramos-Escudero et al., 2019; Chirinos et al., 2013). Due to its high nutritional value, SI is extensively used in food, contributing significantly to human health (Kodahl, 2020). It has gradually been recognized as an extremely valuable crop (Cárdenas et al., 2021). As a crop with huge economic potential for commercial development, SI is being developed in some areas of Southeast Asia, such as Myanmar, Laos, Yunnan Puer, Xishuangbanna and other places in China (Qing et al., 2023).

In addition to SI’s role in food, numerous reports suggest its efficacy in various diseases (Rojanaverawong et al., 2023; Li et al., 2020b; Li et al., 2020a; Thi Phuong Nhung et al., 2023). It has various activities, including antioxidant, anti-inflammatory, lowering blood lipids and blood pressure, improving immunity and memory, etc., (Kim and Joo, 2021a; Kittibunchakul et al., 2023). Therefore, SI can be used to treat cardiovascular diseases, rheumatoid arthritis, diabetes, attention deficit hyperactivity disorders, and inflammation (Lanhua et al., 2021; Wang et al., 2018). In addition, its antioxidation properties make it useful in the cosmetics industry for skin protection and anti-aging purposes (Gonzalez-Aspajo et al., 2015).

Currently, there are different concerns about the safety of natural products, not only compound herbal preparations but also simple herbal products contain many bioactive ingredients that may have toxic effects (Heydari et al., 2022). Therefore, more and more scholars are paying attention to the safety of natural products. As a herbal products, some researchers are also concerned about the safety and toxicity of SI. Some literature suggests that the risk assessment of herbal medicine mainly focuses on the evaluation of drug side effects, toxicity, contraindications, phase interactions, and pharmacokinetic studies (Barrett et al., 1999; Heywood et al., 2024). There are some studies investigating the safety and toxicity of SI, which indicate that consumption of raw saponin seeds may cause toxicity in humans due to the presence of phytotoxins (alkaloids, lectins, and saponins). When using SI seeds, heating is generally required (Kim and Joo, 2021a). There were no signs of morbidity or mortality in mice given deoiled SI cake at a concentration of 2000 mg/kg bw. Gorriti et al. also studied the toxicity of Sacha Inchi oil (SIO) and failed to demonstrate the existence of a median lethal dose (LD50) of SIO. However, the research team predicts that the LD50 may exceed 37 g/kg bw, which represents the highest dose tested in this study (Mhd Rodzi and Lee, 2022). Different methods have been widely used to assess the safety of natural products. These methods include *in vitro* and *in vivo* studies assessing toxicity in different cells and organs (Gonzales and Gonzales, 2014). Some studies have conducted acute toxicity tests, genotoxicity tests and traditional teratogenicity tests on SI to evaluate the toxicity of SI. Research has shown that administering SIO mice (0.9 g/mL) every 4 hours for three consecutive times within 24 h resulted in LD50 values greater than 54 g/kg bw, which is classified as non-toxic according to the acute toxicity classification criteria (Chaopei et al., 2012a). Some studies have also reported on the genotoxicity experiments of SI. The results of the genetic toxicity test showed negative results for different doses of SIO administered through the *Salmonella typhimurium*/mammals microsomal enzyme test (Ames test) (Zeng et al., 2012). There have also been studies exploring the traditional teratogenicity testing of SI. Research has shown that rats were given different doses of SIO for 10 consecutive days starting from the 7th day of conception. The pregnant mice did not show any symptoms of poisoning. A study explored the traditional teratogenicity testing of SI. Research has shown that from the 7th day of conception, rats were given different doses of SIO for 10 consecutive days. At the tested dose, pregnant mice showed no symptoms of toxicity, no abnormalities in organs, and SIO had no maternal toxicity, embryotoxicity, or teratogenicity to pregnant rats. No abnormalities were found in the lungs, liver, kidneys, spleen and other organs of each group of fetal mice. This indicates that at the tested dose, SIO has no maternal toxicity, embryotoxicity, or teratogenicity in pregnant rats (Chaopei et al., 2012b). Research has shown that the most likely adverse reactions associated with SIO are gastrointestinal reactions such as nausea and vomiting, which are relatively common in many drugs such as nonsteroidal anti-inflammatory drugs (Theken et al., 2020). However, studies have shown that the gastrointestinal reactions associated with SIO may be related to its taste. With prolonged use, acceptance of SIO also increases, and this adverse reaction can be alleviated, indicating that SI is suitable for RA patients (Gonzales and Gonzales, 2014). It is worth noting that when using SIO in RA patients, we should also pay attention to the impact of this gastrointestinal adverse reaction on RA patients in the early stages. Patients should take some measures to alleviate gastrointestinal reactions when taking SI related drugs, such as eating after meals or adjusting their diet, neutralizing gastric acid, and reducing discomfort (Lacy et al., 2018). Alternatively, this issue can be addressed by developing dosage form of SI drugs. In summary, existing reports indicate that SI is relatively safe in the treatment of RA. However, current reports have only conducted in-depth research on its acute and subacute toxicity, without evaluating its long-term (chronic) toxicity, and there are no reports on the pharmacokinetics and drug interactions of SI. Therefore, we should further study and explore it in the future.

### 1.2 Rheumatoid arthritis (RA)

Rheumatoid arthritis (RA) is a multifactorial systemic autoimmune inflammatory disease of unknown etiology (Jang et al., 2022). The pathological manifestations of RA include immune cell infiltration, synovial intimal hyperplasia, pannus formation, and destruction of articular cartilage and bone (Jang et al., 2022). Epidemiological studies indicate that RA affects approximately 0.5%–1.0% of the global population (Firestein, 2003; Kaur et al., 2024), with its prevalence increasing almost uniformly since 1990 and peaking between the ages of 35 and 50 years (Almutairi et al., 2021). Currently, the annual incidence of RA is about 3 cases per 10,000 people, and its global prevalence is around 1% (Prasad et al., 2023). RA is more common in women, who have two to three times of risk to develop the disease comparing with men, and its incidence is on a noticeable upward trend (Favalli et al., 2019). Statistical analyses and interpretations of quantitative data indicate that RA is not only a medical but also a growing public health concern (Deane and Holers, 2021). In addition to joint damage, RA affects other tissues and organs (Wu et al., 2022), leading to a range of diseases, increased mortality risk (Figus et al., 2021), and significant healthcare costs.

Although the pathogenesis of RA remains unclear, studies have identified several contributing factors, including genetic susceptibility and environmental risk factors such as smoking (the strongest environmental risk factor for RA), exposure to dust, viral infections, obesity, low socioeconomic status, and gut microbiota alterations (Venetsanopoulou et al., 2022). These factors induce targeted activation of the immune system, leading to the progression of RA. The earliest change in RA is the activation of antigen-dependent T lymphocytes (Yan et al., 2022; Sumida et al., 2024). Subsequently, these T cells differentiate into T helper 17 (Th17) and T helper 1 (Th1) (Yang et al., 2019), which in turn trigger the activation of B cells to produce plasma cells. Concurrently, this process also activates macrophages, neutrophils and synovial cells, resulting in the secretion of large amounts of pro-inflammatory cytokines and metalloproteinases. These pro-inflammatory molecules drive the inflammatory response and bone erosion characteristic in RA (Firestein and McInnes, 2017; Di Matteo et al., 2023).

### 1.3 The main therapeutic drugs for treating RA

At present, the main treatments for RA include nonsteroidal anti-inflammatory drugs (NSAIDs), glucocorticoids, and biological agents (Abbasi et al., 2019). However, these drugs lack specificity and often require long-term, high-dose administration, which can cause serious adverse effects such as gastrointestinal reactions, hepatotoxicity, nephrotoxicity (Guo et al., 2023). Approximately one-third of patients interrupt treatment due to their inability to tolerate these side effects (Zhao et al., 2021).

NSAIDs are one of the mainstream anti-RA drugs in clinical practice, which can effectively alleviate pain and inflammation in RA patients (Nawaz et al., 2021). NSAIDs exert their pharmacological effects by inhibiting cyclooxygenase (COX) and prostaglandins (Richard et al., 2023). However, inhibiting prostaglandins can cause many side effects, such as gastrointestinal discomfort, abdominal pain, diarrhea, dizziness, headache, and other neurological problems, as well as kidney damage and renal failure (Bindu et al., 2020). Glucocorticoids have proven efficacy in controlling disease activity and delaying the progression of joint damage in RA (Doumen et al., 2023). Their primary anti-inflammatory mechanism involves the inhibition of both innate and adaptive immune activity. Nevertheless, they can cause side effects such as obesity, gastrointestinal reactions, nervous system damage, and liver damage (Bruscoli et al., 2022). Long-term use of glucocorticoids, particularly at higher doses, is generally not advisable (Doumen et al., 2023).

Biological agents are a crucial component of modern RA treatment strategies. Biological agents are proteins produced by biotechnology that have inhibitory effects on the body fluids and cellular components of RA. The categories of substances used in RA include tumor necrosis factor-α(TNF-α), interleukin (IL)-1, IL-6, IL-12, IL-17, and IL-23 inhibitors that are effective on cytokines, as well as T lymphocyte activation inhibitors abazil and B lymphocyte depletion rituximab (Fiehn, 2022). They inhibit the humoral and cellular components of rheumatic inflammation (Fiehn, 2022). However, serious side effects, including pneumonia, tuberculosis, and viral infections have been observed during treatment with these molecular-targeted agents (Koike, 2015). These adverse effects can cause significant harm to the human body. Therefore, discovering safe and effective anti-RA drugs from natural plants is a promising approach.

### 1.4 Potential analysis of SI in the treatment of RA

Given the global burden of RA and the growing interest in plant-based preventive and therapeutic solutions, understanding the potential of SI in the treatment of RA, could pave the way for novel therapies. Unlike traditional anti-RA drugs, SI has been consumed as a traditional food in cooked and roasted form (Goyal et al., 2022), which has also drawn considerable interests from pharmacologists due to its anti-inflammatory properties (Thi Phuong Nhung et al., 2023), and its ability to regulate gut microbiota based inflammatory and immune diseases (Li et al., 2020b). SI exerts its anti-inflammatory effects by targeting immune cells, including macrophages and neutrophils, thereby regulating the release of inflammatory cytokines such as TNF-α, IL-6 and IL-1 to control the inflammatory response (Díaz-González and Hernández-Hernández, 2023). Additionally, it plays a role in modulating specific inflammatory pathways (Wang et al., 2022), such as NF-κB, ERK, and IκBα signaling pathways.

For many years, the medical treatment of RA has been basically based on the use of corticosteroids and traditional antirheumatic drugs (DMARDs), such as methotrexate (MTX) (Kerschbaumer et al., 2020). The emergence of biological DMARDs (bDMARDs) has significantly changed the treatment model for RA patients (Smolen et al., 2023). Studies have shown that Filgotinib (a selective JAK 1 inhibitor) (50 mg) as add-on therapy or monotherapy to MTX demonstrated rapid and sustained (to 24 weeks) improvements in health-related quality of life and functional status in patients with active RA (Genovese et al., 2018). But over time, many of these drugs show increased toxicity and decreased efficacy. Etanercept (ETN) is a TNF-α inhibitor used to treat RA. In combined ETN and MTX treatment, 8.3% of patients reported severe infections. All major illnesses that occurred multiple times during the 3 years were: pneumonia, septic arthritis, and postoperative wound infection. During the three-year study, five patients died. Congestive heart failure, autoimmune diseases, demyelinating diseases, and malignancies, are also some of the recent safety concerns (Ejaz et al., 2024). These studies have shown that traditional anti-RA drugs, biologic drugs, and their combination still have major safety issues. Weighing treatment options for patients with RA largely depends on three major factors: efficacy, adverse reaction profile, and cost (Korol and Baumeister, 2023). Although SI has certain adverse reactions, compared with traditional anti-RA drugs, the adverse reactions of SI are relatively mild, and are only accompanied by symptoms such as nausea, and vomiting, and these symptoms improve over time. These symptoms may be caused by its excessively stimulating taste. However, over time, people’s acceptance of SIO may also increase, and this phenomenon may be alleviated (Gonzales and Gonzales, 2014). At the same time, research has also shown that its liver and kidney markers remain unchanged. Some studies have reported that after 60 days, the indicators of urea, and alkaline phosphatase in rats were detected. Compared with the physiological saline control group, the differences were not statistically significant. It shows that SIO is harmless when administered orally to rats for 60 days (Gorriti et al., 2010). There are also research reports that show SIO in rats at different consecutive dose groups. There are also research reports showing that rats are given different doses of SIO. The results showed that the rats grew well, with normal activity, organ coefficients, and pathology. Histological examination showed no biological significance and no significant harmful effects were observed (Zeng et al., 2012). These subacute toxicity experiment indicate that SI is relatively safe at 30 and 60 days and may be used for the treatment of RA. But RA is an autoimmune disease that requires long-term drug treatment. Currently, there have been no studies reporting on its long-term toxicity. Therefore, in future research, further studies should be conducted on the long-term toxicity reactions (teratogenicity, carcinogenicity, and mutagenicity) of SI to further evaluate its safety. SI is relatively safe compared to traditional anti-RA drugs and biological agents. And it indicates that SI has good anti-inflammatory effects, and its safety cost is relatively low. In summary, SI has a certain potential to resist RA.

**FIGURE 1:**
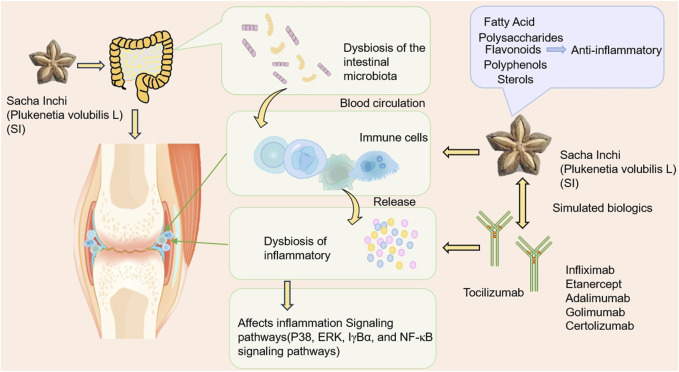
The possible way SI exerts its anti-RA effect. SI may exert anti-RA effects by regulating gut microflora, targeting T cell, neutrophil and macrophage, thereby inhibiting a series of inflammatory cytokines such as IL-6, IL-1, TNF-α, etc.

Based on the pathological process of RA mentioned above, we speculate that SI can comprehensively regulate RA from multiple dimensions and targets. This article provides a multidimensional review of the impact of SI on RA, focusing on the following aspects: modulating gut microbiota, targeting inflammatory cells and associated pathways, and its potential to act similarly to biological antibody drugs, as well as the future application trends of SI active compounds in treating RA. Furthermore, we predict that SI exerts a therapeutic role by acting on the aforementioned pathological links in the pathogenesis of RA. The possible ways in which SI may exert its anti-RA effects are shown in Figure 1.

## 2 Method

**FIGURE 2:**
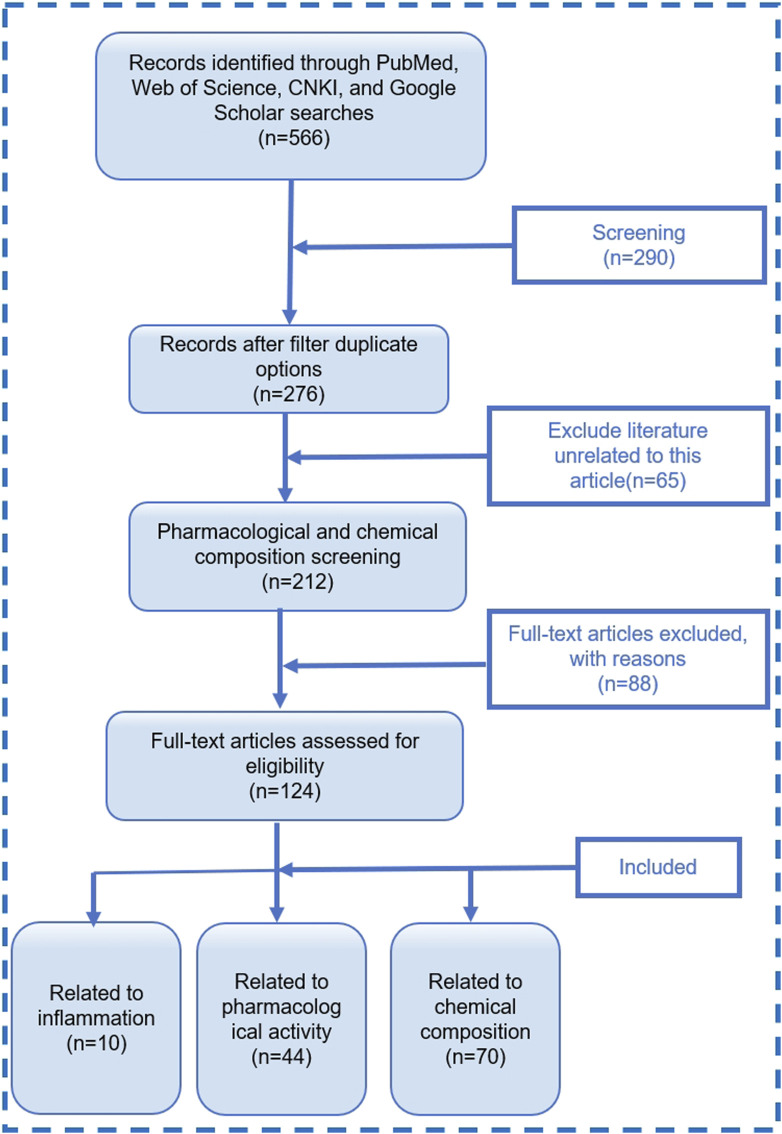
Literature screening process diagram. “with reason” refers to literature that does not match this review.

Literature search was conducted on four major electronic databases, namely, PubMed, Web of Science, China National Knowledge Infrastructure (CNKI), and Google Scholar. Collect relevant information by searching for themes or keywords such as “Plukenetia volubilis Linneo”, “Plukenetia volubilis”, “Sacha Inchi”, “inflammation”, and “rheumatoid arthritis” for the years 2010–2024. A total of 566 articles related to SI were retrieved. After removing duplicates, there were 276 papers, including 212 research papers. Further screening of 124 related articles. Among them, there are 10 articles related to inflammation, 44 articles related to pharmacological activity, and 70 articles related to chemical composition. The literature search process is shown in Figure 2.

## 3 The effect of SI on RA

### 3.1 SI exerts a therapeutic effect on RA by regulating gut microbiota

Although the pathogenesis of RA remains unclear, studies have shown that gut microbiota plays an important role in the development of RA (Xu et al., 2021). Gut microbiota dysbiosis may disrupt gut homeostasis and contribute to the pathogenesis of RA. Some studies suggest that gut dysbiosis may precede arthritis and that local intestinal inflammation can lead to systemic inflammation in genetically predisposed individuals (Tsetseri et al., 2023). This process may trigger the host’s innate immune system and activate the “gut-joint axis”, which exacerbates RA (Xu et al., 2022a). Patients at risk of RA have immune abnormalities, gut microbiota may affect gut immunity by regulating T cell-mediated mucosal immunity (Xu et al., 2020).The gut microbiota interacts with T cells through antigen-specific recognition or signals through toll like receptors (TLRs) and nod like receptors (NLRs). These signals mediate cell induction and function, ensuring the homeostasis of the human immune system. Microbial community types have been shown to be associated with the differentiation of T cells such as Th1, Th2, Th17, and regulatory T (Treg) cells (Shim et al., 2023). Studies have shown significant differences in the gut microbiota of RA patients compared to healthy controls, with an increase in Bacteroidetes, *Escherichia*, and *Shigella*, and a decrease in *Lactobacillus* (L.) (Attur et al., 2022). Oral administration of L. casei or L. acidophilus has been found to reduce arthritic inflammation, pannus formation, and cartilage destruction in rats with adjuvant-induced arthritis (AIA) (So et al., 2008; Paul et al., 2021). Currently, probiotics are used clinically to correct intestinal dysbiosis and reduce the inflammatory cytokine cascade, which has received increasing attentions. It is increasingly recognized that L. and Bifidobacterium may be among the most effective probiotics, with levels of these bacteria significantly reduced in RA patients (Bungau et al., 2021).

It has reported that Sinomenine improves RA by enriching two beneficial species with anti-collagen-induced arthritis (CIA) activities, including L. paracasei and L. casei, which could activate aryl hydrocarbon receptor (AhR) and regulate Th17/Treg balance in CIA rats (Jiang et al., 2023). The gut microbiota can significantly influence the host’s immune system by regulating bone marrow cells such as neutrophils and macrophages. These regulation effects can activate TLRs (Maeda and Takeda, 2017) or NLRs, stimulating T cell differentiation and promoting the expansion of certain pathways that affect RA (Li and Wang, 2021).

Sacha inchi (*Plukenetia volubilis* L.) husk extract (SISE) has been discovered to increase beneficial bacteria such as Akkermansia and L (Li et al., 2020b). This increase in beneficial bacteria reduced common inflammatory indicators, such as C-reactive protein (CRP) L. acidophilus can inhibit the secretion of pro-inflammatory cytokines (IL-1β, IL-6, TNF-α, IL-17 and IL-23) that were mediated by Th17 cells, while increase the secretion of anti-inflammatory cytokines (IL-10) (Vaghef-Mehrabany et al., 2014). Other L. species like L. reuteri, L. casei, L. rhamnosus and L. fermentum inhibit species-specific pro-inflammatory cytokine to alleviate RA (Zhexin Fan et al., 2020). In particular, L. reuteri and L. casei reduce Th1 immune responses, while L. rhamnosus and L. fermentum reduce Th17 immune responses (Zhexin Fan et al., 2020). One strain of L. casei, Shirota was reported to inhibit the occurrence of autoimmune diseases by changing the cytokines produced by antigen-presenting cells, thereby inhibiting the differentiation of lymphocytes into specific subsets of effector T cells (Sánchez et al., 2011). Therefore, the increase of beneficial bacteria can have a relief effect on RA. We thus speculate that the therapeutic effect of SI on RA may partially come from their effects to increase beneficial bacteria, especially *Lactobacillus*.

Moreover, SI can significantly reduce the relative abundance of harmful bacteria like Prevotella copri (Pcopri) (Li et al., 2020a), which cause dysbiosis in RA patients. And SISE can remarkably reduce the abundance of harmful bacterial species of the genera of Parabacteroides, Prevotella and *Bacteroides* (Li et al., 2020a). Pcopri is a major bacterial group associated with dysbiosis in RA patients (Alpizar-Rodriguez et al., 2019). Prevotella stimulates dendritic cells (DCs) to release IL-1β, IL-6 and IL-23 through TLR2, thereby mediating the activation of neutrophils and promoting Th17 cells to produce IL-17. Prevotella directly induces dysfunction in recruited neutrophils, exacerbating inflammation (Larsen, 2017). Pcopri can inhibit the onset of arthritis by stimulating Th1 cells and reducing Th17 response and inflammatory cytokines (i.e., IL-2, IL-17, and TNF-α), thereby treating RA. Pcopri is also related to the production of Th17 cytokines and anti-citrullinated protein antibodies (ACPA) (Drago, 2019), a disease-specific biomarker of RA. Therefore, we speculate that SI can regulate a series of Th1 mediated immune responses by reducing Pcopri, thereby alleviating RA.

**FIGURE 3:**
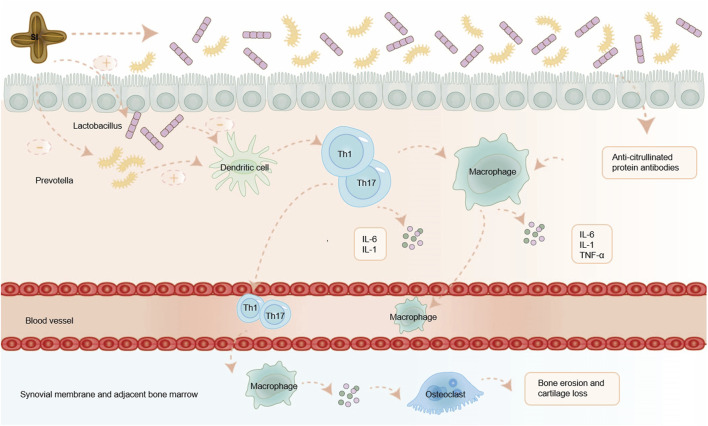
SI exerts anti-RA effects by regulating gut microbiota. SI can alleviate RA by regulating the balance of gut microflora, inhibiting a series of inflammatory immune responses mediated by T cell, and reducing the release of inflammatory factors. IL-1: interleukin-1; IL-6: interleukin-6; TNF-α: tumor necrosis factor-α.

Based on this, we predict that by adding some beneficial bacteria and reducing harmful bacteria, SI can restore Th1 mediated immune inflammation, restore the imbalance of gut dendritic cell in the body, and have a therapeutic effect on RA. Consequently, we anticipate that SI can combat RA by modulating gut microbiota, presenting a viable treatment strategy. The possible mechanism of SI exerting anti-RA effects through gut microbiota is shown in Figure 3.

### 3.2 SI regulates inflammatory response by targeting immune cells

SI not only modulates the gut microbiota, but also inhibits the secretion of pro-inflammatory cytokines mediated by Th1 and Th17 cells, maintain the balance of gut microbiota, and thereby alleviate RA. In the presence of inflammatory signals, neutrophils first infiltrate into the sites of infection, injury, or inflammation. Neutrophils are the main source of reactive oxygen species (ROS) during inflammation (Fresneda Alarcon et al., 2021). Excessive production of ROS drives the inflammatory response by inducing the expression of pro-inflammatory genes (Biswas, 2016). Oxidative stress occurs when there is an imbalance between ROS and reactive nitrogen species (RNS), which causes damage to human tissues through immune-inflammatory responses (Zhou and You, 2022). Therefore, finding effective anti-inflammatory drugs that inhibit ROS production and relieve oxidative stress is considered as a promising strategy for preventing and treating diseases related to chronic inflammation. Antioxidants can mitigate the damage caused by oxidative stress (Zamudio-Cuevas et al., 2022). SI has antioxidant effect and inhibitory effect on matrix metalloproteinase 2 (MMP-2) (Lourith et al., 2023). SI can also improve insulin resistance in type 2 diabetic rats by inhibiting oxidative stress and inflammation (Rojanaverawong et al., 2023). By measuring 2,2-azino-bis-(3-ethylbenzothiazoline-6-sulfonic acid) diammonium salt (ABTS), 2,2-Diphenyl-1-picrylhydrazyl (DPPH), Sacha Inchi oil (SIO) was found to have potential *in vitro* antioxidant activity (Bueno-Borges et al., 2018). Therefore, we predict that SI can reduce oxidative stress and target neutrophils by increasing antioxidant activity, thereby exerting a certain therapeutic effect on RA.

**FIGURE 4:**
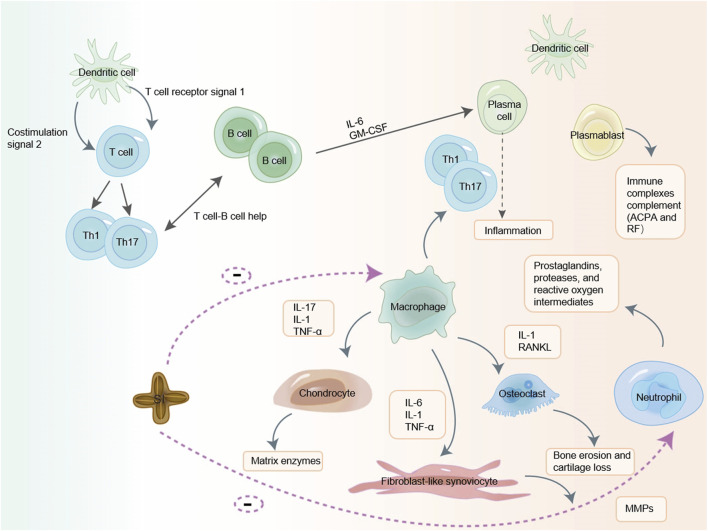
SI may exerts anti-RA effects by targeting immune cells. SI may exert a certain anti-RA effect by targeting T cell, macrophage and neutrophil to reduce cytokines.IL-1: interleukin-1; IL-6: interleukin-6; IL-17: interleukin-17; TNF-α: tumor necrosis factor-α; GM-CSF: granulocyte-macrophage colony-stimulating factor; ACPA: anti-citrullinated protein antibodies; RF: rheumatoid factor; MMPs: matrix metalloproteinases; RANKL: receptor activator of nuclear factor-κB ligand.

Macrophages are one of the immune cells closely related to the occurrence and development of RA (Li et al., 2022). During chronic inflammation such as autoimmune diseases, tissue-resident macrophages fail to solve aggravated inflammation that leads to immune system abnormal activation and damage. And peripheral monocytes are recruited and differentiated into macrophages non-homeostatically in combination with injury-associated signals including pro-inflammatory cytokines, which are further activated and participated in the body’s immune responses (Yang et al., 2023). When an inflammatory process is triggered by the perturbation of tissue homeostasis, bone-marrow derived monocytes that circulate in the blood-stream are attracted to the site of inflammation, through a specific milieu of pro-inflammatory chemokines secreted by resident macrophages, stromal and endothelial cells. At the site of inflammation, monocytes differentiate into macrophages, which cooperate with resident cells for sustaining immunity or promoting resolution of inflammation and tissue regeneration (Viola et al., 2019). These macrophages modulate the immune response not only through direct contact but also by secreting cytokines, which play roles in immunoregulation, tissue remodeling, and process of fibrosis (Dai et al., 2022). In the body, they polarize into different phenotypes and then exert pro-inflammatory or anti-inflammatory effects (Cutolo et al., 2022). Infiltrating macrophages further mediate various inflammatory cells and accelerate inflammation by secreting pro-inflammatory cytokines to promote the production of Th17 cells and stimulate osteoclast differentiation. At the same time, macrophages mediate the chemotaxis and proliferation of endothelial cells, promote the formation of pannus and the infiltration of inflammatory cells, and further expanding the inflammatory response of RA by producing vascular endothelial growth factor (VEGF) (Yang et al., 2023). RAW264.7 cell line is a mouse peritoneal mononuclear macrophage that releases a variety of inflammatory factors (NO, TNF-α, IL-6, IL-1β) after being stimulated by lipopolysaccharide (LPS) (Cui et al., 2021). Studies have shown that SI can reduce the production of pro-inflammatory cytokines IL-6 and TNF-α by macrophages (Jing-xia et al., 2015; Thi Phuong Nhung et al., 2023). Macrophages induce oxidative stress through the release of cytokines (such as TNF-α and IL-1, IL-6, IL-12, IL-15, IL-18, and IL-23), production of reactive oxygen intermediates, nitrogen intermediates, prostaglandins, and matrix-degrading enzymes, as well as through phagocytosis and antigen presentation to exert their effects (Johnny et al., 2016). Numerous studies have shown that SI contains substances such as fatty acids, phytosterols, polyphenols, etc., which have antioxidant effects to improve oxidative stress and have a certain impact on inflammation (Lourith et al., 2023; Bueno-Borges et al., 2018; Rojanaverawong et al., 2023). The mechanism of unsaturated fatty acid antioxidant mainly includes the following aspects. Firstly, unsaturated fatty acids can directly neutralize oxygen free radicals by reacting with them, reducing their damage to cells (Gardner, 1989). Secondly, unsaturated fatty acids can enhance the antioxidant capacity of cells by activating the intracellular antioxidant enzyme system (Ishihara et al., 2019). Finally, unsaturated fatty acids can also inhibit the production of oxygen free radicals by regulating intracellular signaling pathways, thereby reducing the damage of oxygen free radicals to cells (Oppedisano et al., 2020). The antioxidant effect of phytosterol mainly involves the participation of allyl groups on the side chains of sterol compounds in free radical scavenging reactions, which have a certain ability to scavenge oxygen free radicals and thus exert antioxidant effects (Xiaohang et al., 2021; Li et al., 2017). The antioxidant activity of phenolic compounds mainly depends on their chemical structure, especially the number and position of hydroxyl groups and the presence of aromatic rings (Leopoldini M and Toscano, 2011). These compounds act as effective reactants for oxygen reactive substances, reducing and chelating iron ions that catalyze lipid peroxidation. They can also transfer hydrogen atoms to these molecules or provide electrons to free radicals (de Lima Cherubim et al., 2020). Therefore, we hypothesize that SI can target macrophages to reduce inflammatory cytokines such as IL-6 and TNF - α, and exert anti-RA effects by improving oxidative stress. The possible mechanism diagram of SI exerting anti-RA effect by targeting immune cells is shown in Figure 4.

### 3.3 SI can simulate the therapeutic effect of biological antibody drugs on RA

Cytokines is a diverse array of proteins pivotal for regulating inflammation and immunity. Mediate the interaction between cells (Hofer and Campbell, 2016). In RA, cytokines produced by various cell populations within the inflamed synovium are widely acknowledged as central to disease development. Notably, TNF-α and IL-6 play crucial roles in initiating and perpetuating inflammation in RA (Pandolfi et al., 2020). Consequently, blocking the binding of TNF-α or IL-6 to their receptors is a cornerstone of current clinical strategies aimed at managing inflammation and alleviating symptoms (Liu et al., 2021). Modern approaches to RA treatment have introduced biologics, which target inflammatory molecules, cells, and pathways implicated in tissue damage by inhibiting these pivotal cytokines, thereby exerting anti-RA effects (Abbasi et al., 2019). While biologics effectively attenuate the inflammatory response, alleviate symptoms, and control disease progression, they are associated with certain risks. Increased susceptibility to tuberculosis and viral infections is the most common side effects (Curtis et al., 2016), and there may also be an elevated long-term risk of cancer (Bongartz et al., 2006). Moreover, biologics are prohibitively expensive for many patients, limiting their broader application. Therefore, the discovery of natural product-derived drugs capable of ameliorating RA by inhibiting TNF-α and IL-1 represents a significant advancement in RA drug development.

Elevated levels of pro-inflammatory cytokines TNF-α and IL-6 in serum and joints can contribute to excessive bone destruction (Yokota, 2024). Research suggests that SI may exert an anti-inflammatory effect by modulating cytokines, including TNF-α, IL-1β, and others (Guidoni et al., 2019). Clinically, IL-6 inhibitor (tocilizumab) can play a role in treating RA by blocking IL-6 activity (Patel et al., 2023). Cytokines from the interleukin-1 family, such as IL-1α, IL-1β, are notably present in RA and stimulate the activation of various cells like leukocytes, chondrocytes, and osteoclasts. Some studies have shown that polyunsaturated fatty acids have certain antioxidant effects and can exert anti-inflammatory effects by suppressing the secretion of inflammatory cytokines. A report has observed enhanced vitality, lymphocyte stimulation index, and levels of anti-inflammatory cytokine IL-2 and IL-4 in healthy male Wistar rats fed a diet supplemented with SIO compared to those fed a standard diet (Li et al., 2016). In a clinical trial consuming breakfast portions supplemented with SI oil, blood samples taken both fasting and 4 h post-meal showed that SI oil attenuated the increase of IL-6 levels induced by fat intake, suggesting its potential inhibitory effect on IL-6 (Alayón et al., 2019). IL-6 is primarily produced by macrophages in response to pathogens or inflammation-related damage-associated molecular patterns and exerts protective functions by inducing acute phase and immune responses to remove infectious agents and heal damaged tissue. IL-6 also upregulates the expression of receptor activator of nuclear factor kappa B (RANK) ligand (RANKL) on osteoblasts and synoviocytes, leading to osteoclasts differentiation and pannus formation (Pandolfi et al., 2020). Inhibition of IL-6 can mitigate osteoclasts differentiation and pannus formation, thus offering therapeutic benefits for RA. Consequently, SI is anticipated to exhibit anti-RA effects by reducing IL-6 production.

**FIGURE 5:**
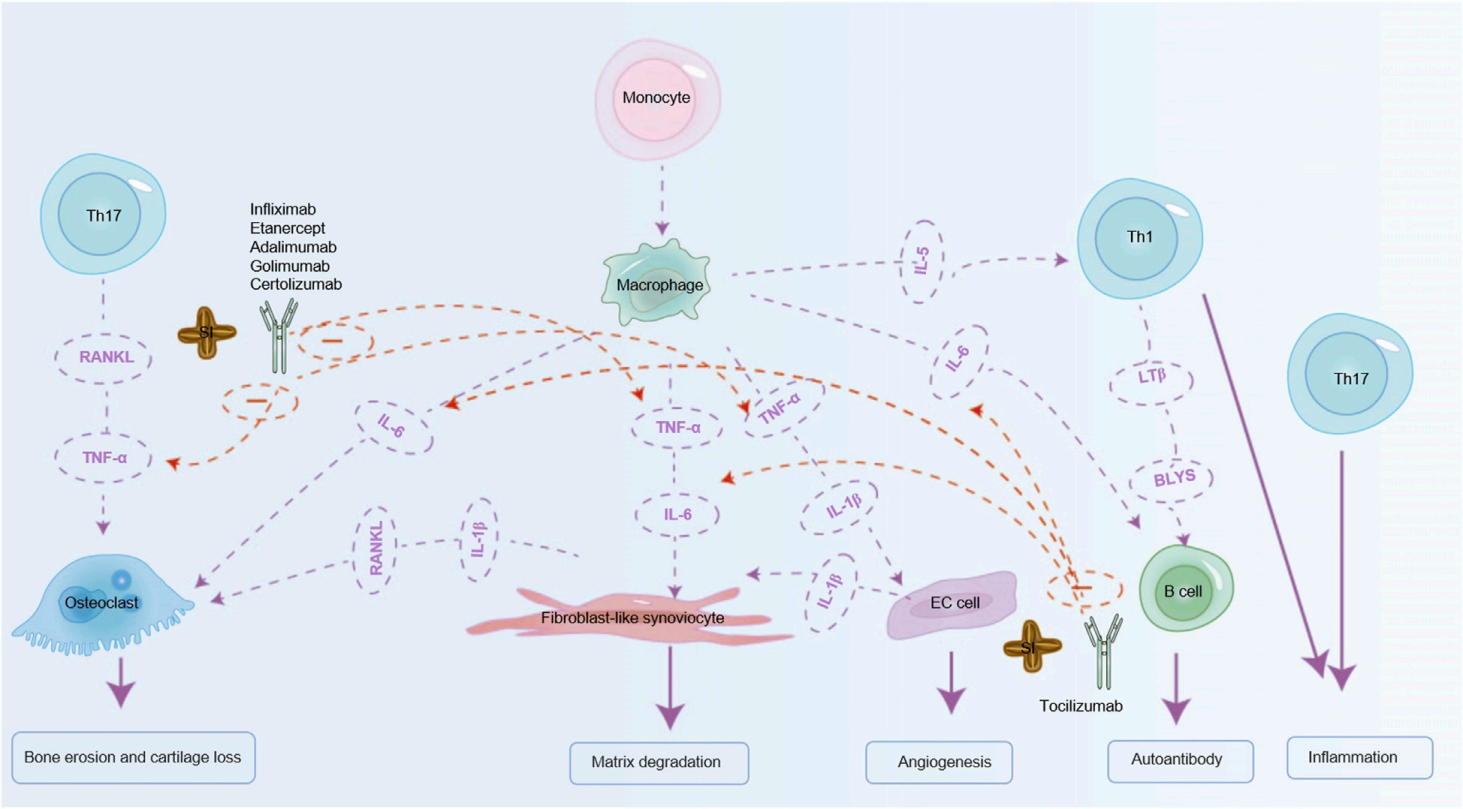
SI can exert anti-RA effects by simulating biological agents. SI can mimic the effects of IL-6 inhibitor (tocilizumab) on RA by inhibiting IL-6 to relieve RA. SI can also simulate TNF-α inhibitors (infliximab, etanercept, adalimumab, golimumab, certolizumab) to relieve RA by inhibiting TNF-α. IL-1β: interleukin-1β; IL-6: interleukin-6; IL-5: interleukin-5; TNF-α: tumor necrosis factor-α; RANKL: receptor activator of nuclear factor-κB ligand; BLYS: B lymphocyte stimulator; LTβ: Lymphotoxin Beta.

TNF-α inhibitors are also widely used clinically, including infliximab, etanercept, adalimumab, golimumab and certolizumab (TNF-α inhibitors), which can treat RA by antagonizing TNF-α (Leone et al., 2023). Initially recognized for its role in tumor necrosis, TNF-α has been implicated in the pathogenesis of autoimmune diseases, demonstrating significant therapeutic efficacy across various autoimmune conditions (Jang et al., 2021). Studies have highlighted the protective role of SI in non-alcoholic fatty liver disease in rats, attributed to its downregulation of TNF-α and IL-6 (Jing-xia et al., 2015). SI has the ability to modulate pro-inflammatory cytokines TNF-α and IL-6 in diabetic rats, ameliorating inflammation and impacting diabetic conditions positively (Rojanaverawong et al., 2023). TNF-α is primarily produced by macrophages and Th1 cells. In RA, TNF-α activates synovial fibroblasts, leading to excessive production of cathepsins and matrix metalloproteinases (MMPs). What ensues is the breakdown of collagen and proteoglycans, leading to cartilage and bone destruction, and joint erosion. Osteoclasts in RA further induce synovial proliferation and angiogenesis (Jang et al., 2021). Therefore, we predict that SI can offer a therapeutic effect on RA by influencing the inflammatory factors TNF-α and IL-6 respectively. Given the established effects of inhibiting TNF-α and IL-6 in RA, researchers have investigated combined inhibition of these cytokines to enhance therapeutic outcomes. Bispecific anti-TNF/IL-6 nanobody compounds or combined therapies have demonstrated superior efficacy compared to monospecific interventions (Biesemann et al., 2023). Notably, SI has been shown to inhibit both TNF-α and IL-6, suggesting potential comparable benefits to bispecific anti- TNF-α/IL-6 nanobody compounds in RA treatment. The mechanism of action of SI simulated TNF-α and IL-6 biologics is shown in Figure 5. Meanwhile, it can also reduce other inflammatory factors and mimic the anti-RA effects of other biological inhibitors. For example, SIO can also inhibit the expression of inflammatory cytokines such as TNF-α, IFN-γ, IL-2, IL-4, and IL-1β in atopic dermatitis mice (Zhang et al., 2024). Compared with the RA model, the experimental group treated with SI leaf extract can significantly reduce TNF-α, IL-1β, IFN-γ, and IL-6, and significantly restore IL-10 levels, thereby exerting a certain anti-RA effect (Thi Phuong Nhung et al., 2023).

### 3.4 SI plays a certain therapeutic role in RA by targeting inflammatory pathways

**FIGURE 6:**
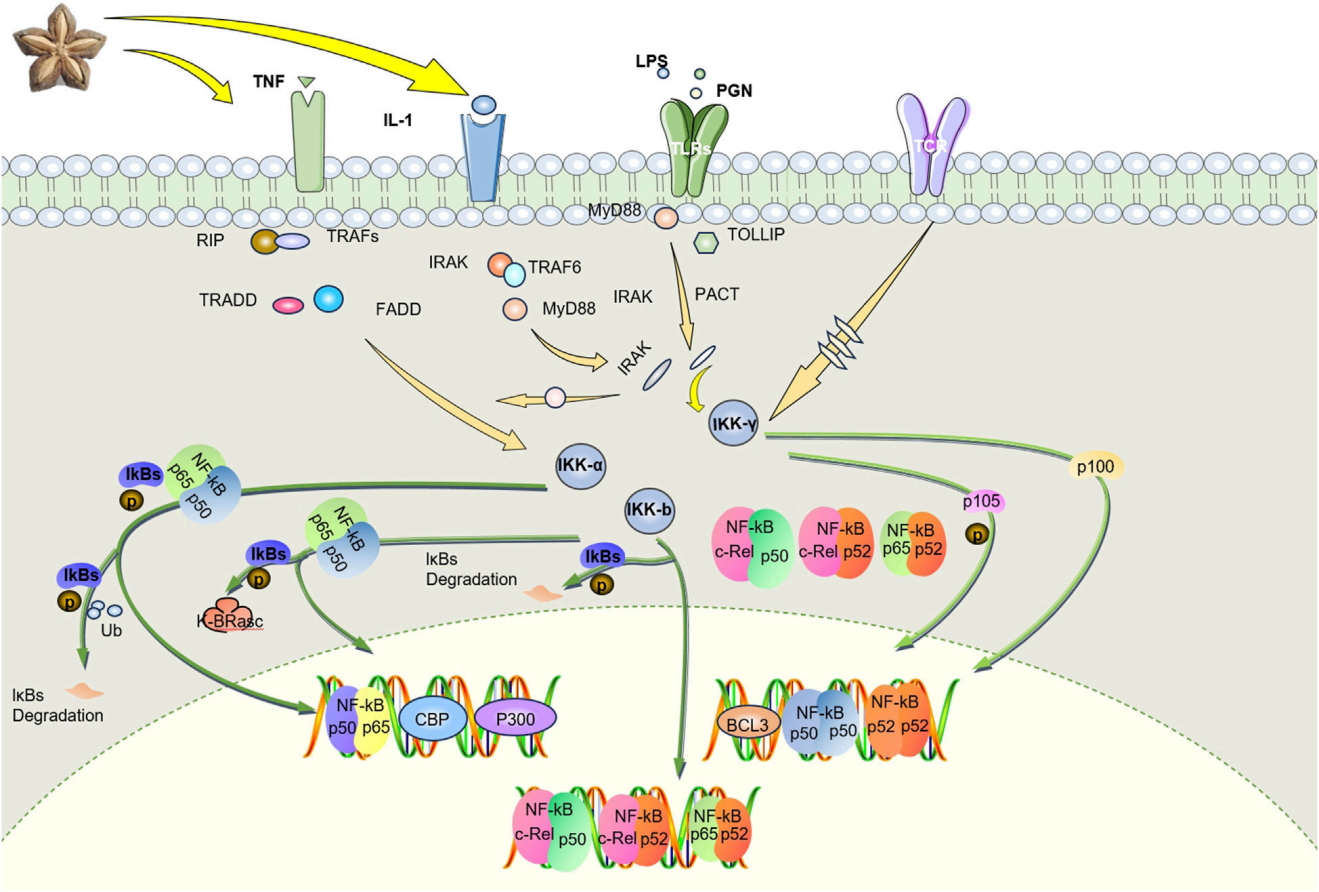
SI targets TNF- α mediate NF-κB pathway exerts anti-RA effects. SI inhibits the proliferation of macrophage by negatively regulating the NF-κB pathway and promotes their apoptosis by playing an anti-inflammatory role. IL-1, interleukin-1; TNF, tumor necrosis factor; LPS, lipopolysaccharide; PGN, peptidoglycan; TLRs, toll like receptors; TCR, T cell receptor; RIP, receptor-interacting protein; TRAFs, tumor necrosis factor receptor associated factors; TRADD, tumor necrosis factor receptor 1 associated death domain protein; FADD, fas-associating protein with a novel death domain; IRAK, interleukin-1 receptor associated kinase; TRAF6, tumor necrosis factor receptor associated factor 6; MyD88, myeloid differentiation primary response gene (88); PACT, photoacoustic computed tomography; TOLLIP, toll-interacting protein; Ikk-α, inhibitor of nuclear factor-κB kinase-α; Ikk-β, inhibitor of nuclear factor-κB kinase-β; Ikk-γ, inhibitor of nuclear factor-κB kinase-γ; IKBs, inhibitor of kappa B.

Numerous studies have demonstrated that certain drugs exert anti-inflammatory effects via modulation of the NF-κB pathway (Kim et al., 2022; Dang et al., 2020). The NF-κB pathway is pivotal in RA pathogenesis and represents a critical target in RA treatment (Zhang et al., 2020; El-Shitany and Eid, 2019). Current literature underscores that inflammatory cytokines such as TNF-α, IL-1β, and IL-6 stimulate NF-κB transcriptional activity, leading to osteoclast activation, enhanced ROS production, and consequent bone resorption (Wei et al., 2019). And research has also shown that SI can reduce the levels of TNF-α, IL-1β, and IL-6 (Thi Phuong Nhung et al., 2023; Alayón et al., 2019; Rojanaverawong et al., 2023; Jing-xia et al., 2015). There are also studies indicating that SIO has inhibitory effects on the protein expression and phosphorylation level of NF-κB, and suppresses the activation of the NF-κB signaling pathway in atopic dermatitis mice (Zhang et al., 2024). Therefore, we predict that SI may further regulate NF-κB signaling pathway by regulating inflammatory factors. Transcriptome analysis has shown that SI can inhibit toll like receptors 4 (TLR4) gene (Wang et al., 2022). TLR 4 gene functions as a uric acid (UA) receptor (Liu-Bryan et al., 2005). When UA is activated by TLR4, its downstream signaling cascade (such as the IKK/IκB/NF-κB pathway) will be released, ultimately leading to induction of pro-inflammatory cytokines/mediators and tissue damage (Zhou et al., 2012). Therefore, we can predict that SI can exert certain anti-inflammatory effects by inhibiting NF-κB signaling pathway based on the downregulation of some genes related to NF-κB signaling. The regulatory effect of SI on NF-κB pathway is shown in Figure 6. Transcriptomics studies have shown that most of the significantly downregulated genes were associated with inflammatory responses, such as regulation of ERK1 and ERK2 cascade, and positive regulation of MAPK cascade. Moreover, it further indicates that SISE group substantially reduced the expression of genes involved in PI3K-Akt signaling pathway, Jak-STAT signaling pathway, TLRs signaling pathway, and NLRs signaling pathway (Wang et al., 2022). Among them, MAPK and PI3K/Akt are also an important pathways in inflammation, and studies have shown that anti-angiogenic effect of Shikonin in RA by downregulating PI3K/AKT and MAPKs signaling pathways (Liu et al., 2020). There are also other studies indicating that some drugs exert anti-RA effects by regulating PI3K/AKT and MAPK signaling pathways (Wang et al., 2023; Tu et al., 2022). At the same time, studies have also explored the effects of SIO on atopic dermatitis, indicating that SIO has inhibitory effects on the protein expression and phosphorylation levels of P38, ERK, NF-κB, and IκBα in their respective signaling pathways, and inhibits the activation of P38, NF-κB, ERK, and IκBα signaling pathways in atopic dermatitis mice (Zhang et al., 2024). We can infer that SIO exerts significant anti-RA effects by regulating the P38, ERK, IκBα, and NF-κB signaling pathways. Therefore, we predict that SI may exert its therapeutic effect on RA by targeting inflammatory pathways.

### 3.5 SI may play a role in some inflammatory and immune diseases by regulating immunity

Many chronic inflammatory diseases are immune-related disorders caused by dysregulated immune responses to autoantigens or environmental antigens (Yang and Cong, 2021). In fact, dysregulated immune responses and imbalances in these mechanisms can lead to tissue damage, inflammation, and autoimmunity (Blanco and Kaplan, 2023). RA is now understood as a chronic immune-mediated disease in which multiple immune cells types and signaling networks become dysfunctional to trigger maladaptive tissue repair processes that lead to organ damage, primarily in joints but also in the lungs and vascular system. The archetype of T cell-mediated inflammation is delayed-type hypersensitivity, also known as type IV hyperresponsiveness, in which macrophages present target antigens to stimulate IFN-γ production and antigen-specific Th1 cells are activated. Th1 cells are widely considered to be the pathogenic T cell subset in RA because they exhibit infiltration of activated CD^4^ T cells and macrophages. The immunopathogenesis of RA and its animal models is often explained by an imbalance between Th1 and Th2 (Yamada, 2023). Through *in vitro* lymphocyte immune experiments, it was found that the polypeptide of SI can promote the proliferation of splenic lymphocytes in mice, with the concentration of 160 μg/mL showing the most significant proliferative effect. The polypeptide of SI can promote the proliferation of splenic lymphocytes within a certain concentration range, which exhibit certain immunoregulatory effects (Rui, 2017). Additionally, Sacha Inchi albumin, concanavalin, and LPS have a synergistic effect on the proliferation of T and B lymphocytes (Fengying, 2016).

Macrophages participate in specific and non-specific immune responses, have phagocytosis, chemotaxis and immunomodulatory functions, and are involved in the occurrence and development of RA (Song et al., 2023). The phagocytic function of macrophages is diminished in autoimmune diseases, thereby inhibiting the clearance of apoptotic cells. The increase in apoptotic cells promotes the production of autoimmune antigens and antibodies and further aggravates inflammation (Yang et al., 2023). Research has shown that the proliferation and phagocytic activity of RAW264.7 macrophages induced by LPS (10 μg/mL) is lower than that of the blank control group, indicating that LPS inhibit the proliferation and phagocytic activity of macrophages. The Sacha Inchi soluble dietary fiber (SISDF) meal can stimulate RAW264.7 macrophages, activate the immune system (Zhao et al., 2022). The active peptide of Sacha Inchi albumin can play a certain immune and anti-inflammatory role by promoting the proliferation of macrophages, promoting the activation of RAW 264.7 macrophages, and improving the phagocytic capacity to exert certain immune and anti-inflammatory effects (Rui, 2017). Studies have also shown that the phagocytic index of the 1000 mg/kg group is higher than that of the control group, suggesting that SIO can significantly promote the phagocytosis of mouse monocytes-macrophages and enhance their immunity. Based on the immune regulatory function of SI mentioned above, although its immune regulation may be less associated with RA, it may play a certain role in other autoimmune diseases.

## 4 Future application trends of SI active compounds in treating RA

### 4.1 Application of SI fatty acids in treating RA

Various nutritional therapies have been proposed for RA, especially those that are rich in omega-3 fatty acids in the diet, which may alleviate inflammation by changing the ratio of omega-6 and omega-3 fatty acids and increasing antioxidants (Schönenberger et al., 2021). Some of the effects of omega-3 are caused by regulating the quantity and type of arachidonic acid produced, while other effects are caused by arachidonic acid independent mechanisms, including effects on intracellular signaling pathways, transcription factor activity, and gene expression (Simopoulos, 2002). According to reports, the anti-inflammatory effects of unsaturated fatty acids include a decrease in the activation of the pro-inflammatory transcription factor nuclear factor kappa light chain enhancer in activated B cells due to the inhibition of the phosphorylation of NF-κB inhibitory subunit IκB under inflammatory stimulation (Djuricic and Calder, 2021; Weatherill et al., 2005; Lee et al., 2001). Inflammasome is a multi-protein complex assembled by natural immune recognition receptors in the cytoplasm, which can mediate the production of various inflammatory mediators such as IL-1 and is crucial for the occurrence of inflammatory reactions (Li et al., 2020c). Omega-3 fatty acids can inhibit the activation of nucleotide-binding oligomerization domain-like receptor protein 3(NLRP3) inflammasome, reduce the secretion of key inflammatory factor IL-1β, and alleviate inflammation (Yan et al., 2013). Inhibiting these inflammatory factors can alleviate osteoclast differentiation and the formation of vascular opacities, thereby providing therapeutic benefits for RA. Research shows that the purest component in SI is fatty acids (Goyal et al., 2022). Indicating that SI has a certain level of effectiveness. One study conducted a randomized, double-blind, placebo-controlled study in which 30 subjects received 10 or 15 mL of SIO daily for 4 months (Gonzales and Gonzales, 2014). Studies have shown that most of the adverse reactions experienced by subjects in the first few weeks of taking SIO were nausea and vomit. The acceptability of SIO was 37.5%, but as the number of days of taking SIO increased, these adverse reactions gradually disappeared, and the acceptability of SIO gradually increased to 81.25%–93.75% (Gonzales and Gonzales, 2014). And no serious effects of SIO were observed, and biochemical markers of liver and kidney function remained unchanged. This indicates that SI has a certain level of safety compared to traditional anti RA drugs. The content of unsaturated fatty acids in SIO reaches more than 93%, including oleic acid (ω-6, 5.3%–9.1%), linoleic acid (ω-6, 33.4%–38.6%) and α-linolenic acid (ω-3, 43.6%–50.8%) (Rodríguez et al., 2022), which can reduce total cholesterol, triglycerides, low-density lipoprotein and high-density lipoprotein levels, and possess anti-inflammatory and antioxidant effects (Gonzales and Gonzales, 2014; Garmendia et al., 2011). These active ingredients are commonly found in animal and human skin and have functions such as improving skin microcirculation, antibacterial, anti-inflammatory, antioxidant and anti-aging (Chandrasekaran and Liu, 2014). Both ω-6 and ω-3 fatty acids regulate the release of pro-inflammatory cytokines such as TNF-α, IL-6, and IL-1 to control the inflammatory response, promote the production of collagen fiber tissue cells, effectively enhance the healing of skin wounds, and reduce inflammation (Guidoni et al., 2019). Therefore, we predict that the fatty acid components in SI have potential applications in RA, and may play a significant role in combating RA through mechanisms such as antioxidant activity, and regulation of inflammatory factors.

### 4.2 Application of SI polyphenols in treating RA

In recent years, with the rise of the development and utilization of natural products, plant polyphenols have emerged as the most valuable source of natural antioxidants (Ji et al., 2020), and play a pivotal role in antioxidative and anti-inflammatory processes (de Lima Cherubim et al., 2020). Polyphenols can play a certain role in various diseases through anti-inflammatory effects (Rana et al., 2022). There are studies indicating that SI contains a certain amount of polyphenols (Ramos-Escudero et al., 2021), which may have the ability to chelate with Fe^2+^. Polyphenolic compounds have the ability to provide electrons and can easily form coordination bonds with Fe^2+^ to chelate Fe^2+^, and SIO has strong ability to remove OH, thus exerting an antioxidant effect (Xiaohang et al., 2021). Among these, resveratrol stands out for its beneficial role in preventing and managing chronic inflammatory conditions. Resveratrol can inhibit the expression of TLR and pro-inflammatory genes (Malaguarnera, 2019). LC-MS/MS analysis of SI revealed the presence of resveratrol, suggesting its potential as an effective anti-inflammatory component in SI, warranting its further research and development (Yanti et al., 2022). Research reports that SISE is rich in polyphenols and flavonoids, with five flavonoids and nine polyphenolic compounds detected, such as quercetin, naringenin, hesperidin, kaempferol, isorhamnetin, gallic acid, 4-Hydroxybenzoic acid, p-coumaric acid, caffeic acid, vanillic acid (Kittibunchakul et al., 2022b). The scavenging effects of SISE on hydroxyl free radicals and DPPH free radicals are better than those of tert-butylhydroquinone (TBHQ), vitamin C (VC) and butylated hydroxytoluene (BHT), suggesting its excellent antioxidant capacity (Jin-ming et al., 2021). The young and mature leaves of SI also contain phenolic substances as detected by high-performance liquid chromatography (HPLC), including three flavonoids (kaempferol, apigenin and isorhamnetin), and eight phenolic acids (gallic acid, vanillic acid, 4-hydroxybenzoic acid, caffeic acid, p-coumaric acid, syringic acid, sinapinic acid, and ferulic acid) (Kittibunchakul et al., 2022a). Among them, studies have shown that the anti-inflammatory function of quercetin comes from its antioxidant effect, which can reduce nitric oxide (NO), superoxide dismutase (SOD), glucose-6-phosphate (The expression of oxidative stress markers) and glutathione (GSH) (Lin et al., 2020). Naringenin can significantly reduce the overproduction of IL-1β and TNF-α, and can also attenuate oxidative stress (Huang et al., 2019). SI can inhibit the expression of pro-inflammatory mediators by targeting the NF-κB and MAPK pathways (Cho and Shaw, 2024). Studies have shown that hesperidin significantly inhibit the gene expression of inflammatory mediators induced by LPS and reduce the protein levels of matrix metalloproteinase-3 (MMP3), matrix metalloproteinase-9 (MMP9) and matrix metalloproteinase-13 (MMP13), inhibit the polarization of macrophages toward M1. And hesperidin inhibition of the PI3K/AKT signaling pathway may show a therapeutic effect in the progression of RA (Qi et al., 2019). Studies have proven that kaempferol inhibits the migration and invasion of fibroblast-like synoviocytes (FLSs) in RA by blocking the activation of MAPK pathway, reducing the severity of arthritis in mice with CIA (Pan et al., 2018). Studies have shown that isorhamnetin treatment can significantly reduce leukocyte recruitment and excessive secretion of IL-6 and monocytes chemoattractant protein-1 (MCP-1). Isorhamnetin mainly affects Nrf2/Keap1 pathway and significantly alleviates the inflammatory response in chronic obstructive pulmonary disease (COPD) mice (Xu et al., 2022b). The anti-inflammatory mechanism of gallic acid mainly involves the MAPK and NF-κB signaling pathways, which weakens the inflammatory response by reducing the release of inflammatory cytokines, chemokines, adhesion molecules and cell infiltration (Bai et al., 2021). Among them, the flavonoid apigenin also has a good anti-inflammatory effect. Apigenin can improve the symptoms of arthritis in freund complete adjuvant (FCA) rats. Its anti-inflammatory mechanism may be through reducing cytokines (IL-1β, IL-6 and TNF-α) and inhibiting the P2X7/NF-κB signaling pathway (Chang et al., 2015). Caffeic acid also inhibits serum oxidative stress by reducing lipid peroxides and NO and increasing reduced glutathione in arthritic animals to reduce joint damage (Fikry et al., 2019). P-Coumaric acid can significantly inhibit the levels of inflammatory cytokines and chemokines (TNF-α, IL-1β, IL-6 and MCP-1), reduce fibroblast-like synoviocyte ogenic factors (RANKL and TRAP) in arthritic rats, and inhibit the expression of pro-inflammatory cytokines (TNF-α, IL-1β, IL-6 and IL-17) and inflammatory enzymes (iNOS and COX-2). Furthermore, p-Coumaric acid truncated osteoclastogenesis by regulating RANKL/OPG imbalance in arthritic rats (Neog et al., 2017). Ferulic acid is able to reverse changes in biochemical parameters and inflammatory markers, such as CRP and rheumatoid factor (RF). Furthermore, study found that complete freund adjuvant (CFA)-induced arthritis triggered TNF-α secretion, increased JAK2 levels, and decreased transforming growth factor-β (TGF-β) levels in tissue homogenates. This change improved significantly after administration of ferulic acid, which might improve arthritis by inhibiting the JAK/STAT pathway (Zhu et al., 2020). Ferulic acid significantly inhibits IL-17-mediated toll-like receptor 3 (TLR3) and cysteine-rich angiogenesis in FLS by inhibiting the IL-17/IL-17RA/STAT-3 signaling cascade. The expression of inducer 61 (Cyr61), IL-23, and granulocyte-macrophage colony-stimulating factor (GM-CSF) (Ganesan and Rasool, 2019). Sinapic acid suppresses immune responses by inhibiting IκB kinase (IKK). Sinapic acid reduces inflammation and oxidative stress by downregulating IKK, thereby alleviating rheumatoid arthritis (Wang et al., 2021). Protocatechuic acid inhibits the proliferation, invasion and migration of RA-FLSs in a dose-dependent manner. Protocatechuic acid treatment also inhibited the expression of MMP-3 and MMP-13 in RA-FLSs, as well as the secretion of inflammatory cytokines TNF-α, IL-1β, and IL-6. In addition, Protocatechuic acid treatment significantly induced apoptosis in RA-FLSs cells, inhibited the activation of NF-κB signaling, decreased p-p65 expression and increased IκBα expression. At the same time, Protocatechuic acid significantly reduced the phosphorylation levels of Akt and mammalian target of rapamycin (mTOR) in RA-FLSs. Protocatechuic acid has an inhibitory effect on RA-FLSs by inhibiting the NF-κB and Akt/mTOR signaling pathways (Wu et al., 2020). Chlorogenic acid can inhibit the production of B cell activating factor (BAFF) in serum and the production of serum TNF-α, and inhibit TNF-α induced BAFF expression and MH7A cell apoptosis in a dose-dependent manner. A DNA binding site for the transcription factor NF-κB in the BAFF promoter region is required for this regulation. Research results indicate that Chlorogenic acid may be used as a new therapeutic agent targeting BAFF to treat RA (Fu et al., 2019). Chlorogenic acid effectively controlled total T cell (CD3) and differentiated T cell (CD4 and CD8) counts. Chlorogenic acid inhibits Th1 cytokines in a very significant manner but increases Th2 cytokines (Chauhan et al., 2012). Therefore, we predict the phenolic components in SI can improve oxidative stress and anti-inflammatory effects through antioxidant effects and exert anti-RA effects.

### 4.3 Application of SI phytosterols in treating RA

Phytosterol is a natural active substance known as the “key to life” (Zhang et al., 2023). Among them, β-sitosterol, the most common and abundant phytosterol, is widely used in medicine, nutraceuticals, and cosmetics and has high nutritional value as well as immunomodulatory and anti-inflammatory properties (Schönenberger et al., 2021). β-Sitosterol exerts certain anti-inflammatory effects by inhibiting the recruitment of neutrophils, which are the main source of reactive oxygen species (Li et al., 2018). And β-Sitosterol can reduce oxidative stress through enhanced antioxidant activity and pro-inflammatory factors. Additionally, it inhibits NF-κB and extracellular signal-regulated kinase (ERK) mitogen-activated protein kinase (MAPK) signaling, thereby reducing the inflammatory response of mouse microglia induced by LPS (Sun et al., 2020). β-Sitosterol can also reduce mRNA expression of inflammatory factors, such as IL-6 and TNF-α (Sun et al., 2020). Sterols are natural antioxidant components abundant in vegetable oils, which can effectively delay lipid peroxidation. Studies have revealed that the sterol content in SIO is higher than that of other common vegetable oils (Li et al., 2018), endowing it with robust oxidative stability (Jiangyue et al., 2021). HPLC analysis of a crude sterol extract from SIO identified β-sitosterol and stigmasterol as its primary detectable components. The allyl group on the side chain of sterol compounds in vegetable oils participates in free radical scavenging reactions, enhancing their ability to scavenge O^2-^ (Xiaohang et al., 2021). Consequently, we anticipate that phytosterols, particularly β-sitosterol, may hold promise in combating RA.

### 4.4 Application of SI polysaccharides in treating RA

Plant polysaccharides have important effects on anti-inflammatory processes, exhibiting certain antioxidant activity, and can regulate macrophages function by controlling the secretion of cytokines, exerting certain immune regulatory effects (Wang et al., 2019). The SI soluble dietary fiber (SISDF) showcases promising antioxidant and immune activities, suggesting potential for the development of natural food antioxidants and anti-inflammatory health products. Studies employing techniques such as ion chromatography, gel chromatography, and fourier transform infrared spectroscopy have revealed that SISDF is a macromolecular polysaccharide with pyranose as the main chain. SISDF has been found to enhance the proliferation and phagocytic activity of RAW264.7 macrophages, and increase the secretion levels of NO, IL-1β, IL-6, and TNF-α factors in RAW264.7 macrophages (Zhao et al., 2022). Interestingly, another study showed that a novel water-soluble polysaccharide (PVLP-1) induced the proliferation of RAW264.7 cells and increased the expression of inflammatory factors IL-6, TNF-α, and IL-1β, thus enhancing immune function (Tian et al., 2020). Therefore, we predict that SI polysaccharides can alleviate RA by regulating oxidation and immunity. And its polysaccharide components may also play a certain role in inflammatory and immune diseases by regulating immunity. The compounds in SI that may have an impact on RA are listed in Table 1.

**TABLE 1:** Compounds in SI that may have an impact on RA.

Active ingredient	Form	Pharmacological action	Source	References
Fatty acid	oleic acid (5.3%–9.1%) linoleic acid (33.4%–38.6%) alpha linolenic acid (43.6%–50.8%)	Antioxidant activity, improvement of oxidative stress and regulation of inflammatory factors ↓pro-inflammatory cytokines such as TNF - α IL-6 and IL-1 ↑collagen fiber tissue cell production, anti-aging ↓total cholesterol, triglycerides, low-density lipoprotein and high-density lipoprotein levels, antioxidant properties	Oil	Liu et al. (2020), Wang et al. (2023), Tu et al. (2022), Yang and Cong (2021)
Flavonoids	Quercetin (1.72 ± 0.06 mg/100 g)	Antioxidant ↓lipid peroxides (LPO) ↓nitric oxide (NO) ↓superoxide dismutase (SOD) ↓glucose-6-phosphate (G6PD) ↓ glutathione (GSH)	Shell	Kittibunchakul et al. (2022b), Lin et al. (2020)
Flavonoids	Naringenin (29.21 ± 0.17 mg/100 g)	↓inflammatory factors (IL-1β, TNF-α) ↓oxidative stress NF-κB and MAPK pathway	Shell	Kittibunchakul et al. (2022b), Huang et al. (2019), Cho and Shaw (2024)
Flavonoids	Hesperidin (23.92 ± 1.55 mg/100 g)	↓MMP3、MMP9 and MMP13 ↓Polarization of M1 PI3K/AKT pathway	Shell	Kittibunchakul et al. (2022b), Qi et al. (2019)
Flavonoids	Kaempferol (12.63 ± 0.45 mg/100 g)	↓migration and invasion of FLSs MAPK pathway	Shell Leaf	Kittibunchakul et al. (2022b), Pan et al. (2018)
Flavonoids	Isorhamnetin (0.27 ± 0.01 mg/100 g)	↓IL-6,MCP-1 Nrf2/Keap1 pathway	Shell Leaf	Kittibunchakul et al. (2022b), Kittibunchakul et al. (2022a), Xu et al. (2022b)
Flavonoids	Apigenin (10.76 ± 0.13 mg/100 g)	↓p65, TTR, and RAGE ↓maturity and migration of dendritic cells (DCs)	Leaf	Kittibunchakul et al. (2022a), Chang et al. (2015)
Phenolic acid	Gallic acid (49.13 ± 2.67 mg/100 g)	↓expression of pro-inflammatory genes in RA FLS regulating Th17/Treg cell imbalance	Shell Leaf	Kittibunchakul et al. (2022b), Kittibunchakul et al. (2022a), Bai et al. (2021)
Phenolic acid	Caffeic acid (16.21 ± 0.10 mg/100 g)	↓Phosphorylation of IκB and IκB kinase ↓IL-6 and TNF-α ↓Lipid peroxides, nitric oxide ↑Elevated reduced glutathione ↓Oxidative stress	Shell Leaf	Kittibunchakul et al. (2022b), Kittibunchakul et al. (2022a), Fikry et al. (2019)
Phenolic acid	p-Coumaric acid (148.74 ± 2.46 mg/100 g)	↓TNF-α、IL-1β、IL-6 and MCP-1 ↓RANKL and TRAP ↓iNOS and COX-2 adjusting RANKL/OPG pathway imbalance	Shell Leaf	Kittibunchakul et al. (2022b), Kittibunchakul et al. (2022a), Neog et al. (2017)
Phenolic acid	Ferulic acid (5.71 ± 0.09 mg/100 g)	↓Toll like receptor 3 (TLR-3), cysteine rich angiogenesis inducer 61 (Cyr61), IL-23, granulocyte macrophage colony-stimulating factor (GM-CSF) JAK/STAT pathway IL-17/IL-17RA/STAT-3 pathway	Shell Leaf	Kittibunchakul et al. (2022b), Kittibunchakul et al. (2022a), Zhu et al. (2020), Ganesan and Rasool (2019)
Phenolic acid	Sinapic acid (17.41 ± 0.40 mg/100 g)	↓IκB kinase (IKK)	Shell Leaf	Kittibunchakul et al. (2022b), Kittibunchakul et al. (2022a), Wang et al. (2021)
Phenolic acid	Protocatechuic acid (2.8 ± 0.67 mg/100 g)	↓MMP-3 and MMP-13 ↓TNF-α, IL-1β, IL-6 phosphorylation levels of Akt and mTOR NF-κB and Akt/mTOR signaling pathways	Shell	Kittibunchakul et al. (2022b), Wu et al. (2020)
Phenolic acid	Chlorogenic acid (2.16 ± 0.12 mg/100 g)	↓expression of B cell activating factor (BAFF) and apoptosis of MH7A cells ↓Th1 cytokines NF-κB pathway	Shell	Kittibunchakul et al. (2022b), Fu et al. (2019), Chauhan et al. (2012)
Polyphenols	Resveratrol (l2.467 ±0.134 μg/100 g)	↓expression of TLR and pro-inflammatory genes	Oil	Malaguarnera (2019), Yanti et al. (2022)
Sterols	β–sitosterol (435–563 μg/g) Stigmasterol (346–456 μg/g)	Anti-inflammation and antioxidant properties ↓IL-6 and TNF-α	Oil	Lu et al. (2023), Chandrasekaran and Liu (2014), Yanti et al. (2022), Kittibunchakul et al. (2022b)
Polysaccharides	PVLP-1	Antioxidation and immune regulation ↑immunologic function	Oil	Yang and Cong (2021), Gonzales and Gonzales (2014)

## 5 Advantages, limitations and future directions

This review highlights comprehensively on the possible mechanisms of SI in treating RA, indicating that SI as a natural plant, holds great promise in the development of anti-RA drugs. Approved by the Chinese Ministry of Health as a new resource food in 2013, SIO is primarily used as an edible oil and is also added to processed food as an additive (Jiayi et al., 2013). Because SI is rich in protein, the newly developed seed powder is widely used in fast food to enhance nutritional value. Its application in beverages and snack foods has also been reported (Bai, 2021). SI leaves are mainly used in tea drinks, such as health tea, offering health benefits by eliminating harmful substances from the body and boosting immunity. SI also shows promise in alleviating diabetes, hypertension, and some cardiovascular diseases, with applications in various other conditions (Abd Rahman et al., 2023; Li et al., 2020b; Rojanaverawong et al., 2023). SI is a new national resource food, with its by-products holding significant economic and medicinal value. The market prospects are broad, carrying substantial economic value. In clinical practice, anti-RA drugs taken by RA patients can cause certain harm to human body (Guo et al., 2023; Bindu et al., 2020). Therefore, it is a very feasible method to develop SI as an anti-RA drug. However, the current researches on anti-RA mechanisms of SI are quite limited, and no safety report, warranting follow-up preclinical and clinical studies to ascertain SI’s application in RA. However, some related studies have also shown that SI is relatively safe, indicating that LD50 values cannot be achieved at doses as high as 37 g/kg bw. Although consuming SI raw can cause some adverse reactions such as vomiting, and nausea, these adverse reactions will weaken over time, indicating its relative safety. Although there are relatively few studies on SI presently, it has shown a therapeutic effect on inflammation. Yet, there’s a lack of in-depth research on its pharmacological mechanisms at the gene and protein levels, necessitating further investigation into SI’s anti-RA mechanisms. Additionally, due to few ingredients discovered in SI, the active SI ingredients will be a focus of future research. We have predicted the therapeutic and improvement potential of each component in SI for RA. However, it is still unclear how the active ingredients in SI work alone and function. Therefore, in future research, further attention should be paid to the components in SI, and the truly effective components for RA in SI should be identified through methods such as component separation and identification. Pharmacological experiments should be conducted to verify whether they work through a single component or multiple components working together, and how these components work specifically. In summary, SI has excellent anti-inflammatory activity and may have therapeutic effects on RA through gut microbiota, targeting immune cells, regulating inflammatory factors, and some signaling pathways. These may become beneficial factors for RA patients, and through SI, RA may be alleviated. Meanwhile, RA patients also have some risk factors. Firstly, SI can cause gastrointestinal discomfort, and whether RA patients can accept this gastrointestinal reaction. Secondly, whether long-term use of SI will cause damage to certain tissues and organs has not been evaluated. Therefore, when developing SI related drugs, long-term (chronic) toxicity should be further evaluated, and clinical trials should be conducted when developing related products to further assess whether RA patients can accept this gastrointestinal adverse reaction and its related safety.

## 6 Conclusion

This review highlights the significant potential and application prospects of SI as a natural anti-inflammatory drug in RA. SI can regulate the production of inflammatory cytokines (TNF-α, IL-6 and IL-1) by targeting immune cells such as T cells, macrophages, and neutrophils, thereby impacting various pathways including the NF-κB pathway, PI3K-Akt signaling pathway, toll-like receptor signaling pathway, chemokine signaling pathway, Jak-STAT signaling pathway, and nod-like receptor signaling pathway, ultimately exerting an anti-RA effect. Moreover, we also reviewed some ingredients in SI that may play a role in RA, focusing on the fatty acids, polysaccharides, polyphenols, and phytosterol active compounds in SI. These active compounds have a positive impact on RA through antioxidant and anti-inflammatory effects. Among them, unsaturated fatty acids have been the most extensively studied components. Linoleic acid and α-linolenic acid hold considerable potential for treating RA. Although flavonoids are less abundant in SI, apigenin, one of its components, may also contribute to its anti-RA effects. Beta-sitosterol among phytosterols plays an important role in anti-inflammatory processes and thus contributes to its anti-RA effects. Studies have indicated that the polysaccharides in SI possess certain anti-inflammatory effects, but it is not yet clear which component is responsible. Therefore, in the next step, an in-depth study of anti-RA components in SI should be conducted. These remarkable effects position SI as a promising herbal products and complementary therapy in combating RA. Consequently, SI holds significant potential and promising application prospects in RA.
